# Improving macromolecular models of challenging ligands through combined QM/MM X-ray refinement

**DOI:** 10.1063/4.0000838

**Published:** 2025-10-27

**Authors:** Margarita A Tararina, Lance M Westerhoff, Oleg Borbulevych, Matt Pokross, David A Critton

**Affiliations:** 1 Bristol Myers Squibb, Princeton, NJ, USA; 2 QuantumBio, Inc., State Collete, PA, USA

## Abstract

Conventional structural refinement relies on fixed stereochemical restraints for all components within the protein-ligand complex, using pre- determined parameters such as bond lengths, angles, and torsions in the unbound ligand conformation. This approach can be challenging for non-standard modalities like covalent modifiers and macrocycles, where conformational flexibility is inherent to their mechanism of action, and the bound vs. unbound states differ significantly. To address this limitation and achieve a more complete model, we employ an automated molecular refinement approach that replaces stereochemical restraint gradients with quantum-mechanic/molecular mechanic (QM/MM) gradients in standard refinement packages, Phenix and Buster. In this context, we present user test cases in QM/MM structure refinement using QuantumBio’s DivCon plugin with XModeScore to explore potential ligand states and evaluate binding modes based on ligand strain energy and the z-score of difference density.

